# Supplementary material for: Severe infections emerge from commensal bacteria by adaptive evolution
Source: eLife. 2017 Dec 19;6:e30637. doi: 10.7554/eLife.30637 (PMC5736351; doi:10.7554/eLife.30637)
Supplement: Supplementary file 3. — Neutrality indices (NIs, 41,42) were calculated as the odds ratio of the number of protein-altering to synonymous variants among B-class versus C/D-class variants. These tests are less powerful than the Poisson regression likelihood ratio tests used to detect gene or gene set enrichment of protein-altering B-class variants (Table 3); we present them to demonstrate that the direction of enrichment was consistent with adaptation (NI > 1). To mitigate the reduced power, we calculated the expected numbers of protein-altering B-class variants from the numbers of protein-altering C/D-class variants, synonymous B-class variants and synonymous C/D-class variants by pooling them across all genes. This was justified by the absence of evidence for within-patient recombination and lack of enrichment signals among synonymous variants and C/D class protein-altering variants. A one-tailed Poisson test in R (R Core Team, 2015) was used to test NI > 1 (significant NIs at p<0.05 in bold). [file elife-30637-supp3.docx]

| Gene group | | No. protein-altering  B-class variants | |  | Cumulative length  of genes (kb) | |  | Expt. no. protein-altering  B-class variants | |  | Neutrality index  95% C.I. lower bound | |  | Neutrality index point estimate | |  | Significance  (-log_10_ one-tailed *p*) | |  |
| --- | --- | --- | --- | --- | --- | --- | --- | --- | --- | --- | --- | --- | --- | --- | --- | --- | --- | --- | --- |
| Locus | |  |  |  |  |  |  |  |  |  |  |  |  |  |  |  |  |  | |
|  | *agrA* | 5 |  |  | 0.7 |  |  | 0.11 |  |  | 18.40 |  |  | **46.71** |  |  | 6.97 |  | |
|  | *clfB* | 5 |  |  | 2.6 |  |  | 0.39 |  |  | 5.02 |  |  | **12.73** |  |  | 4.25 |  | |
|  | Total | 289 |  |  | 2363.8 |  |  | 354.38 |  |  |  |  |  |  |  |  |  |  | |
|  |  |  |  |  |  |  |  |  |  |  |  |  |  |  |  |  |  |  | |
| BioCyc Gene Ontology (*48*) | |  |  |  |  |  |  |  |  |  |  |  |  |  |  |  |  |  | |
|  | Cell wall | 18 |  |  | 30.9 |  |  | 4.65 |  |  | 2.50 |  |  | **3.87** |  |  | 5.69 |  | |
|  | Cell adhesion | 13 |  |  | 17.2 |  |  | 2.58 |  |  | 2.98 |  |  | **5.03** |  |  | 5.47 |  | |
|  | Pathogenesis | 31 |  |  | 112.5 |  |  | 16.89 |  |  | 1.33 |  |  | **1.84** |  |  | 2.88 |  | |
|  | Total | 288 |  |  | 2359.3 |  |  | 354.38 |  |  |  |  |  |  |  |  |  |  | |
|  |  |  |  |  |  |  |  |  |  |  |  |  |  |  |  |  |  |  | |
| SAMMD Expression Pathway | | *Down-regulated* | *Up-regulated* |  | *Down-regulated* | *Up-regulated* |  | *Down-regulated* | *Up-regulated* |  | *Down-regulated* | *Up-regulated* |  | *Down-regulated* | *Up-regulated* |  | *Down-regulated* | *Up-regulated* | |
|  | Ovispirin-1 (*50*) | 40 | 7 |  | 121.2 | 142.9 |  | 19.36 | 22.82 |  | 1.56 | 0.14 |  | **2.07** | 0.31 |  | 4.57 | 0.00 | |
|  | Temporin L (*50*) | 42 | 14 |  | 125.1 | 156.1 |  | 19.98 | 24.93 |  | 1.60 | 0.34 |  | **2.10** | 0.56 |  | 4.93 | 0.00 | |
|  | *rsp* (*51*) | 27 | 1 |  | 61.1 | 13.7 |  | 9.76 | 2.19 |  | 1.95 | 0.02 |  | **2.77** | 0.46 |  | 5.38 | 0.05 | |
|  | *agrA* (RN27) (*52*) | 9 | 30 |  | 41.0 | 85.0 |  | 6.54 | 13.58 |  | 0.72 | 1.59 |  | 1.38 | **2.21** |  | 0.67 | 4.09 | |
|  | VISA-vs-VSSA (Mu50 vs N315) (*53*) | 0 | 17 |  | 0 | 34.4 |  |  | 5.49 |  |  | 1.97 |  |  | **3.10** |  |  | 4.21 | |
|  | VISA-vs-VSSA (Mu50 vs Mu50-P) (*53*) | 0 | 17 |  | 0 | 36.7 |  |  | 5.86 |  |  | 1.85 |  |  | **2.90** |  |  | 3.88 | |
|  | VISA-vs-VSSA (isolate pair 2) (*54*) | 14 | 3 |  | 26.9 | 59.7 |  | 4.29 | 9.51 |  | 1.97 | 0.09 |  | **3.26** | 0.32 |  | 3.81 | 0.00 | |
|  | *sarA* (RN27) (*52*) | 6 | 23 |  | 49.9 | 57.7 |  | 7.96 | 9.21 |  | 0.33 | 1.71 |  | 0.75 | **2.50** |  | 0.09 | 4.03 | |
|  | *agrA* (UAMS-1 OD 1.0) (*55*) | 0 | 5 |  | 0 | 2.7 |  |  | 0.42 |  |  | 4.64 |  |  | **11.78** |  |  | 4.09 | |
|  | Pine-Oil Disinfectant-Reduced-Susceptibility (*56*) | 17 | 5 |  | 36.4 | 23.6 |  | 5.81 | 3.77 |  | 1.86 | 0.52 |  | **2.93** | 1.33 |  | 3.92 | 0.49 | |
|  | Total | 275 | |  | 2093.5 | |  | 334.37 | |  |  |  |  |  |  |  |  |  | |

**Table S3.** Neutrality indices show signals of adaptation among the genes, gene ontologies and expression pathways most significantly enriched for protein-altering B-class variants. Neutrality indices (NIs, *41,42*) were calculated as the odds ratio of the number of protein-altering to synonymous variants among B-class versus C/D-class variants. These tests are less powerful than the Poisson regression likelihood ratio tests used to detect gene or gene set enrichment of protein-altering B-class variants (Table 3); we present them to demonstrate that the direction of enrichment was consistent with adaptation (NI > 1). To mitigate the reduced power, we calculated the expected numbers of protein-altering B-class variants from the numbers of protein-altering C/D-class variants, synonymous B-class variants and synonymous C/D-class variants by pooling them across all genes. This was justified by the absence of evidence for within-patient recombination and lack of enrichment signals among synonymous variants and C/D class protein-altering variants. A one-tailed Poisson test in R (*87*) was used to test NI > 1 (significant NIs at *p* < 0.05 in bold).
