# Supplementary material for: Severe infections emerge from commensal bacteria by adaptive evolution
Source: eLife. 2017 Dec 19;6:e30637. doi: 10.7554/eLife.30637 (PMC5736351; doi:10.7554/eLife.30637)
Supplement: Supplementary file 5. — We counted the number of protein-altering variants in these genes within patients, and compared to the number in long-term asymptomatic carriers. p-Values calculated using Fisher’s exact test. *Variant totals are different for SAMMD pathways (rsp, agrA, sarA) and BioCyc ontologies (cell wall, cell adhesion, pathogenesis) because pathway information is available for a different number of loci in each database. [file elife-30637-supp5.docx]

| Gene Ontology or Expression Pathway (Loci with protein-altering B_D_-class variants within patients) | Number of variants* | | *p*-value |
| --- | --- | --- | --- |
|  | Within patients | Within carriers |  |
| AgrA locus (SAR2126) | 3/156 | 0/115 | n.s. |
| Rsp transcriptional pathway (spa, SAR0143, clfA, SAR1014, SAR1745, ureA, ureG, SAR2427, fnbA, clfB, sasA, SAR2763) | 16/147 | 0/109 | 0.0001 *** |
| SarA transcriptional pathway (SAR0109, spa, SAR0211, pyrAA, SAR1397, agrC, agrA, SAR2245, SAR2420, SAR2430, hlgB, fnbA, arcC, sasA, lip) | 20/147 | 1/109 (agrC) | 0.0001 *** |
| AgrA transcriptional pathway (spa, SAR0211, pyrAA, SAR1397, sucA, SAR1466, hemL, agrC, agrA, SAR2430, hlgB, hlgC, clfB, arcC, sasA, lip) | 21/147 | 1/109 (agrC) | <0.0001 *** |
| Cell wall (spa, clfA, fnbA, clfB, sasA) | 9/156 | 0/115 | 0.01 * |
| Cell adhesion (clfA, fnbA, clfB) | 6/156 | 0/115 | 0.04 * |
| Pathogenesis (spa, SAR0115, SAR280, SAR0464, SAR0739, saeR, clfA, ebh, rot, SAR2035, SAR2448, hlgA, hlgB, fnbA, clfB, sasA) | 21/156 | 2/115 (ebh) | 0.0006** |

**Table S5**. For all ontologies showing enrichment in within-patient B_D_-class variants, we identified the genes with variants contributing to the signal. We counted the number of protein-altering variants in these genes within patients, and compared to the number in long-term asymptomatic carriers. P values calculated using Fisher’s exact test. *Variant totals are different for SAMMD pathways (*rsp, agrA, sarA*) and BioCyc ontologies (cell wall, cell adhesion, pathogenesis) because pathway information is available for a different number of loci in each database.
