# Supplementary material for: Severe infections emerge from commensal bacteria by adaptive evolution
Source: eLife. 2017 Dec 19;6:e30637. doi: 10.7554/eLife.30637 (PMC5736351; doi:10.7554/eLife.30637)
Supplement: Transparent reporting form [file elife-30637-transrepform.docx]

***eLife’s* transparent reporting form**

We encourage authors to provide detailed information *within their submission* to facilitate the interpretation and replication of experiments. Authors can upload supporting documentation to indicate the use of appropriate reporting guidelines for health-related research (see [EQUATOR Network](http://www.equator-network.org/%20)), life science research (see the [BioSharing Information Resource](https://biosharing.org/)), or the [ARRIVE guidelines](http://www.plosbiology.org/article/info:doi/10.1371/journal.pbio.1000412) for reporting work involving animal research. Where applicable, authors should refer to any relevant reporting standards documents in this form.

If you have any questions, please consult our Journal Policies and/or contact us: [editorial@elifesciences.org](mailto:editorial@elifesciences.org).

**Sample-size estimation**

- You should state whether an appropriate sample size was computed when the study was being designed
- You should state the statistical method of sample size computation and any required assumptions
- If no explicit power analysis was used, you should describe how you decided what sample (replicate) size (number) to use

Please outline where this information can be found within the submission (e.g., sections or figure legends), or explain why this information doesn’t apply to your submission:

Supplementary Materials, Materials and Methods, page 1: We prospectively sampled the largest possible number of patients meeting the study criteria: “105 patients with severe *S. aureus* infections for whom the organism could be cultured from both admission screening nasal swab and clinical sample were identified from the microbiological laboratories of hospitals in Oxford and Brighton, England” [line 2]. Prospective sampling was essential because usually a single colony is stored in freezer collections, thus discarding diversity in the nose and infection site, which then makes it impossible to distinguish substitutions between nose and infection site populations from polymorphisms within them. For each sample (nose or infection site) “we picked five colonies per sample (twelve during the pilot phase involving nine patients)” [line 11] a choice informed by our previous work (Young, Golubchik *et al* 2012 doi:10.1073/pnas.1113219109; Golubchik, Batty *et al* 2013 doi:10.1371/journal.pone.0061319) and the pilot phase, consideration of which led us to choose “five colonies per site as a compromise between power to distinguish within-site polymorphisms from true between-site substitutions and the cost of whole genome sequencing. Under a standard neutral model with low mutation rate, five colonies per sample is expected to confer 91% power to correctly distinguish within-site polymorphisms from between-site substitutions, compared to 0% power with one colony per sample” [line 20].

R code:

```
# What proportion of identified mutant alleles are correctly attributed as polymorphisms?
# Pr(i>0 & i<n|i>0) = Pr(i>0 & i<n)/Pr(i>0) = int_f Pr(i>0 & i<n|f) p(f) / int_f Pr(i>0) p(f)
# where i and f are the count and frequency of the mutant allele and n the sample size
f0 = function(x) 1/x; # Frequency density of a neutral mutant allele
pcor = function(n) integrate(function(x) f0(x)*(1-dbinom(0,n,x)-dbinom(n,n,x)),0,1)$value/ integrate(function(x) f0(x)*(1-dbinom(0,n,x)),0,1)$value
round(100*pcor(5)); # 91
round(100*pcor(1)); # 0
```

**Replicates**

- You should report how often each experiment was performed
- You should include a definition of biological versus technical replication
- The data obtained should be provided and sufficient information should be provided to indicate the number of independent biological and/or technical replicates
- If you encountered any outliers, you should describe how these were handled
- Criteria for exclusion/inclusion of data should be clearly stated
- High-throughput sequence data should be uploaded before submission, with a private link for reviewers provided (these are available from both GEO and ArrayExpress)

Please outline where this information can be found within the submission (e.g., sections or figure legends), or explain why this information doesn’t apply to your submission:

Supplementary Material, Materials and Methods, page 2: “We sequenced 62 genomes in duplicate, a technical replication rate of 5.1%; no genetic discordancies were detected within duplicates” [line 58].

Supplementary Material, Materials and Methods, page 5: “Sequencing reads uploaded to short read archive (SRA) under BioProject PRJNA369475. RNA-Seq data relating to isolate from P005 (aka ‘patient S’) previously submitted under BioProject PRJNA279958” [line 206].

**Statistical reporting**

- Statistical analysis methods should be described and justified
- Raw data should be presented in figures whenever informative to do so (typically when N per group is less than 10)
- For each experiment, you should identify the statistical tests used, exact values of N, definitions of center, methods of multiple test correction, and dispersion and precision measures (e.g., mean, median, SD, SEM, confidence intervals; and, for the major substantive results, a measure of effect size (e.g., Pearson's r, Cohen's d)
- Report exact p-values wherever possible alongside the summary statistics and 95% confidence intervals. These should be reported for all key questions and not only when the p-value is less than 0.05.

Please outline where this information can be found within the submission (e.g., sections or figure legends), or explain why this information doesn’t apply to your submission:

Results pages 4-8, Figures 2, S2, S3, S4, Figure legends S5, S6, Tables 2, 3, S4: All results of statistical analyses are supported by corresponding *p*-values and, where appropriate, summary statistics and sample sizes.

Supplementary Material, Materials and Methods, page 4. Sections titled “***Gene enrichment analysis***”, “***Gene set enrichment analysis***” and “***GSEA multiple testing correction***” detail statistical analysis methods.

Supplementary Material, Materials and Methods, page 5. Section titled “***omegaMap analysis***” details further statistical analysis methods.

(For large datasets, or papers with a very large number of statistical tests, you may upload a single table file with tests, Ns, etc., with reference to sections in the manuscript.)

**Group allocation**

- Indicate how samples were allocated into experimental groups (in the case of clinical studies, please specify allocation to treatment method); if randomization was used, please also state if restricted randomization was applied
- Indicate if masking was used during group allocation, data collection and/or data analysis

Please outline where this information can be found within the submission (e.g., sections or figure legends), or explain why this information doesn’t apply to your submission:

Supplementary Materials, Materials and Methods, page 1: Every set of colonies sampled from a clinical infection site was matched by a set of colonies sampled from the nose of the same patient. This study design builds in robustness to potential confounders by matching disease-causing and nose-colonizing bacteria within the same patients. “Clinical samples comprised 55 blood cultures and 50 pus, soft tissue, bone or joint samples. The bacteria sampled and sequenced from one patient (‘patient S’, P005 in this study) have been previously described (Das, Lindemann *et al* 2016 doi:10.1073/pnas.1520255113). Five individuals had both blood and another culture-positive clinical sample; we focus analysis on the blood sample” [line 7].

**Additional data files (“source data”)**

- We encourage you to upload relevant additional data files, such as numerical data that are represented as a graph in a figure, or as a summary table
- Where provided, these should be in the most useful format, and they can be uploaded as “Source data” files linked to a main figure or table
- Include model definition files including the full list of parameters used
- Include code used for data analysis (e.g., R, MatLab)
- Avoid stating that data files are “available upon request”

Please indicate the figures or tables for which source data files have been provided:

The following supplementary tables provide detailed additional data on the colonies and genetic variants described in this study.

**Table S1**. List of all cultures included in the site, the site of infection (and any known source if bloodstream), number of isolates sequenced from each site, ST or CC by in silico MLST, number of variants found at each site and the mean pair-wise difference comparing isolates.

**Table S2**. List of all variants found within patients with *S. aureus* disease, location on shared reference (MRSA252), or position and reference genome name and accession number if variant could not be localized on MRSA252. Each variant is described by the alleles found, its location in gene, the predicted effect on gene product and the location of the variant on the phylogenetic tree.

**Table S3**. List of all variants found within long term asymptomatic carriers, location on shared reference (MRSA252), or position and reference genome name and accession number if variant was not localized on MRSA252. Each variant is described by the alleles found, its location in gene and the predicted effect on gene product.
